# Comprehensive Analysis of Neurotoxin-Induced Ablation of Dopaminergic Neurons in Zebrafish Larvae

**DOI:** 10.3390/biomedicines8010001

**Published:** 2019-12-28

**Authors:** Michael Kalyn, Khang Hua, Suzita Mohd Noor, Chee Ern David Wong, Marc Ekker

**Affiliations:** 1 Department of Biology, Faculty of Science, University of Ottawa, ON K1N 6N5, Canada; michael.t.kalyn@gmail.com (M.K.); khanghua88@gmail.com (K.H.); 2 Department of Biomedical Science, Faculty of Medicine, University of Malaya, Kuala Lumpur 50603, Malaysia; suzita@um.edu.my (S.M.N.); davidwce89@gmail.com (C.E.D.W.)

**Keywords:** Parkinson’s disease, neurotoxins, dopaminergic neurons, ventral diencephalon, locomotor, zebrafish

## Abstract

Neurotoxin exposure of zebrafish larvae has been used to mimic a Parkinson’s disease (PD) phenotype and to facilitate high-throughput drug screening. However, the vulnerability of zebrafish to various neurotoxins was shown to be variable. Here, we provide a direct comparison of ablative effectiveness in order to identify the optimal neurotoxin-mediated dopaminergic (DAnergic) neuronal death in larval zebrafish. Transgenic zebrafish, Tg(*dat*:eGFP), were exposed to different concentrations of the neurotoxins MPTP, MPP^+^, paraquat, 6-OHDA, and rotenone for four days, starting at three days post-fertilization. The LC_50_ of each respective neurotoxin concentration was determined. Confocal live imaging on Tg(*dat*:eGFP) showed that MPTP, MPP^+^, and rotenone caused comparable DAnergic cell loss in the ventral diencephalon (vDC) region while, paraquat and 6-OHDA caused fewer losses of DAnergic cells. These results were further supported by respective gene expression analyses of *dat*, *th*, and *p53*. Importantly, the loss of DAnergic cells from exposure to MPTP, MPP^+^, and rotenone impacted larval locomotor function. MPTP induced the largest motor deficit, but this was accompanied by the most severe morphological impairment. We conclude that, of the tested neurotoxins, MPP^+^ recapitulates a substantial degree of DAnergic ablation and slight locomotor perturbations without systemic defects indicative of a Parkinsonian phenotype.

## 1. Introduction

Parkinson’s Disease (PD) is a progressive and debilitating neurodegenerative disease in which clinical hallmarks comprise motor and cognitive impairments. Pathologically, motor symptoms in humans arise due to the progressive and irreversible loss of dopaminergic (DAnergic) neurons within the substantia nigra [1]. Although the etiology of PD remains ambiguous, it is likely due to the cumulative result of genetic and environmental insult. Familial PD has been linked to mutations in genes, however, this can only explain 15–30% of cases while the majority of cases are sporadic [2]. It is believed that exposure to environmental toxins is widely associated with idiopathic PD [3].

Animal models contribute to our knowledge of PD progression and help explore possible therapeutics. Chemo-ablative models using neurotoxic compounds such as 1-methyl-4-phenyl-1,2,3,6-tetrahydropyridine (MPTP), 1-methyl-4-phenyl-pyridinium (MPP^+^), 6-hydroxydopamine (6-OHDA), rotenone, and paraquat have been used to study the molecular mechanisms of DAnergic neurodegeneration in mammals [4,5,6,7,8]. The zebrafish (*Danio rerio*) has proven to be a robust in vivo vertebrate animal model for the studies of various neurodegenerative diseases [9,10] and high-throughput drug screening [11] due to their large clutch size and transparent ex utero embryogenesis. With ~87% of their genome similar to humans, zebrafish also display a variety of conserved molecular and pathological pathways [12]. In particular, a proportion of the DAnergic system in zebrafish (primarily in the ventral diencephalic region) is postulated to be functionally homologous to that of the nigrostriatal pathway in mammals [13,14]. Dopaminergic projections in zebrafish are fully formed by 3–4 days post-fertilization (dpf) [12,15] compared to E12.5 observed in mice [16], E20 in rats [17], and by embryonic day 73 in macaques [18]. The rapid neurodevelopment and optical transparency throughout embryogenesis in zebrafish allow for facilitated study of DAnergic-related diseases such as PD.

MPTP is the most widely used neurotoxin to induce DAnergic neuronal loss and PD-associated locomotor phenotypes in many animal models [4,19,20]. MPTP is lipophilic and it is metabolized by astrocytes (glia) into its active toxic form, MPP^+^, by monoamine oxidase B (MAO-B). MPP^+^ enters DAnergic neurons through the dopamine transporter (*dat*) where it can act on complex I of the electron transport chain (ETC), resulting in the inhibition of ATP synthesis, mitochondrial respiration, and in the promotion of superoxide radical production [7,20].

Paraquat is the most predominantly used commercial herbicide [21,22]. Recent epidemiological studies have outlined the increased risk of developing PD following extensive exposure to paraquat [23]. This divalent cation undergoes redox cycling and its presence results in enhanced oxidative-stress within DAnergic mitochondria [24]. However, exposure to paraquat has been shown to yield inconsistent reports in the literature. This is particularly evident in zebrafish studies, where some have outlined a severe neurodegenerative impact of paraquat on dopamine and serotonin protein levels [25,26], while others observed no obvious phenotypes [10]. This variable effect extends to behavioral symptoms following treatment, where some groups observed significant motor impairment [27], while others report no fluctuations in any swimming activity parameter [10]. These results may be the consequence of varying treatment regimens. Paraquat-induced Parkinsonian-related phenotypes in vivo warrant further investigation.

6-OHDA is a synthetically hydroxylated dopamine compound that is taken up into catecholaminergic neurons through dopamine or norepinephrine transporters [28,29]. 6-OHDA exposure is commonly co-administered with a norepinephrine transporter inhibitor to increase the specificity of neurotoxicity to DAnergic neurons [30,31]. Although 6-OHDA has been shown to be effective in inducing DAnergic neuronal loss in a wide variety of mammals from mice, cats, dogs, monkeys, and rats [5], few studies have shown the sensitivity of zebrafish to 6-OHDA.

Likewise, rotenone is commonly used as an industrial insecticide and as piscicide, a compound used to eradicate fish [32]. Rotenone’s hydrophobic nature allows for the easy penetrance of astrocyte and neuronal cellular membranes [33,34]. The mechanism of action of rotenone is similar to MPTP, through the impairment of complex I of the ETC. Additionally, through nuclear translocation, rotenone leads to the activation of NF-κB and the subsequent DNA fragmentation to result in cellular damage [35]. The toxic nature of rotenone to fish makes it difficult to determine an optimal dose that results in efficient DAnergic ablation with low mortality. Rotenone exposure studies in zebrafish have yielded conflicting results [10,36]

To date, the susceptibility of zebrafish to neurotoxic drugs remains contradictory. This may be due to varying concentrations, duration of exposure, and initial time of treatment which typically occurs at 24 h post-fertilization (hpf), before the full DAnergic neuronal system and blood–brain barrier (BBB) have developed [15]. In addition, behavioral assays and methods of determining DAnergic neuronal loss vary greatly between studies. Most studies primarily utilize TH as a marker for detecting DAnergic cells in either in situ hybridization or whole mount immunostaining approaches. The use of a transgenic model, Tg(*dat*:*eGFP*) [37] that expresses enhanced green fluorescent protein (eGFP) under the control of the dopamine transporter (*dat*) cis-regulatory element in zebrafish, allows for live visualization of DAnergic neurons. In this study, we provide a comprehensive comparison of PD-associated neurotoxins and determine the optimal concentration required to induce maximal decrease of DAnergic neurons along with PD-like locomotor phenotypes.

## 2. Materials and Methods

### 2.1. Zebrafish Care and Husbandry

Transgenic zebrafish of the Tg(*dat*:*eGFP*) line [37] were used in this study. Zebrafish were maintained at 28.5 °C under a 14-h light/10-h dark cycle. Embryos were collected by natural spawning and raised in E3 embryo media (13 mM NaCl, 0.5 mM KCL, 0.02 mM Na_2_HPO_4_, 0.04 mM KH_2_PO_4_, 1.3 mM CaCl_2_, 1.0 mM MgSO_4_, and 4.2 mM NaHCO_3_). Embryos were screened using fluorescence microscopy and eGFP^+^ larvae were sorted accordingly. We expressed the embryonic stages in days post-fertilization (dpf). The University of Ottawa Animal Care Committee approved all animal care procedures, which were conducted under the Animal Care and Veterinary Service guidance in accordance with the recommendations of the Canadian Council for Animal Care following ethical code BL-2081.

### 2.2. Environmental Toxin Preparation and Exposure

At 3 dpf, 20 larvae were sorted and placed into each well of a 6-well plate in a solution volume of 3 mL. De-chorionated larvae were treated at 3 dpf until 7 dpf. Room temperature (~23 °C) was chosen for exposures due to an increase of rotenone-associated neurotoxicity observed at 28.5 °C [38] and to ensure all treatment groups are exposed to the same conditions. Five different concentrations were chosen to determine an accurate sub-lethal dosage. The exposure gradient followed an increasing half log series. Solutions were changed daily and replaced with freshly prepared ones. Toxins were discarded in accordance to current safety protocols. All larvae were co-treated with 0.2 M phenylthiourea (PTU), a pigmentation inhibitor, to facilitate confocal live imaging. Previous studies have shown that PTU does not impair *th* or *dat* expression throughout embryonic and larval neurodevelopment [13].

1-methyl-4-phenyl-1,2,3,6-tetrahydropyridine hydrochloride (MPTP; C_12_H_15_N · HCl, Product: M0896, CAS: 23007-85-4, Sigma, Oakville, ON, Canada) was reconstituted in distilled water to a stock concentration of 500 mM. MPTP was further diluted to the working concentrations of 0.25 mM, 0.5 mM, 1.0 mM, 1.5 mM, and 2.5 mM. MPP^+^ iodide (≥98%, C_12_H_12_IN, Product: D048, CAS: 36913-39-0, Sigma) was re-constituted in distilled water to a stock concentration of 4.86 mM. MPP^+^ was further diluted to the working concentrations of 0.025 mM, 0.05 mM, 0.075 mM, 0.1 mM, and 0.5 mM. Methyl viologen dichloride hydrate (Paraquat; ≥98%, C_12_H_14_Cl_2_N_2_ · xH_2_O, Product: 856177, CAS: 75265-73-0, Sigma) was reconstituted with sterile distilled water at a concentration of 100 mM, then diluted into 25 mM, 10 mM, 5 mM, 3 mM, and 1 mM working dosages. 6-Hydroxydopamine hydro-chloride (6-OHDA; ≥97%, (HO)_3_C_6_H_2_CH_2_NH_2_· HCL, Product: H4381, CAS: 28094-15-7, Sigma) was dissolved in and co-administered with 1% ascorbic acid to prevent precipitation and preserve molecular stability at a concentration of 100µm. The solution was further diluted to the working concentrations of 30µM, 10µM, 3µM, and 1µM. Controls for 6-OHDA were exposed to a 0.01% ascorbic acid solution. Rotenone (≥95%, C_23_H_22_O_6_, Product: R8875, CAS: 83-79-4, Sigma) was prepared in 100% dimethyl sulfoxide DMSO at a concentration of 100 mM, then serially diluted up to 1,000,000-fold in 10% water-diluted DMSO to working concentrations of 5 nM, 10 nM, 25 nM, 50 nM, and 100 nM. Rotenone controls were exposed to a 0.01% DMSO solution. All treatments were conducted in the dark as MPP^+^, 6-OHDA, and rotenone are photosensitive.

### 2.3. Live Confocal Imaging

At 7 dpf, treated zebrafish larvae were transferred from their respective exposure media to fish water, anaesthetized with 3× tricaine, and live-mounted dorsal side up on slides using a 1% low-melting point agarose solution. Larvae were continuously re-hydrated while mounted using system water to ensure survival. Imaging was conducted using the Nikon A1 confocal microscope with a 25× water immersion objective. Larvae were scanned using the laser at a wavelength of 488 nm to excite eGFP. Images were obtained in a 2–3 µm interval Z-stack that was processed and compiled to produce a three-dimensional image. The total cell numbers for clusters 8, 12, and 13 were determined in 3-D to avoid repeated counts of the same cell and by three independent researchers in a blinded fashion to remove bias. Maximum intensity projection images used for the study were produced using the NIS-Elements software (Nikon, Mississauga, ON, Canada).

### 2.4. RNA Isolation and qRT-PCR

RNA was extracted from five pools of 10 pestle-homogenized whole larvae using TRIzol (InVitrogen, ThermoFisher, Waltham, MA, USA). Extractions were done according to the manufacturer’s protocol. RNA integrity and purity was determined using gel electrophoresis and the NanoDrop 1000 Spectrophotometer (ThermoFisher, Waltham, MA, USA). Samples with clear 18S, 28S bands and an absorbance ratio of 1.8–2.1 were used for cDNA synthesis. RNA was reverse-transcribed using the iScript™ cDNA Synthesis Kit (Life Science Research, Bio-Rad, St Laurent, Qc, Canada) in accordance with the manufacturer’s protocol. qRT-PCR reactions were composed of 5 μL SsoFast™ EvaGreen^®^ Supermix (Bio-Rad), 0.4 μL reverse primer, 0.4 μL forward primer, 0.2 μL nuclease-free water and 4 μL cDNA. Reactions were done in triplicates using the Bio-Rad CFX96 instrument. Normalized quantification of the number of *th1*, *dat*, and *p53* transcripts was achieved through the comparative Cq method using three reference genes*; tyrosine 3-monooxygenase/tryptophan 5-monooxygenase activation protein, zeta polypeptide* (*ywhaz*), *ribosomal protein l13a* (*rpl13a*), and *elongation factor 1 alpha* (*ef1a*). Oligonucleotide primers are listed in Table 1.

### 2.5. Swimming Activity

Following neurotoxin treatment, 7 dpf larval zebrafish from each treatment group (6-OHDA, Paraquat, Rotenone, MPP^+^, and MPTP) were removed from their exposure media and monitored for their swimming activity. Individual larvae were transferred to E3 embryo media, placed in a 6-well plate, and allowed to acclimate in ambient light for 15 min prior to recording. The activity was recorded using the Zebralab software and the Zebrabox tracking system (ViewPoint Life Science, Lyon, France). The tracking system consists of infrared illumination, LED lights, and a mounted camera for swimming recording under dark and light conditions. The parameters of larval swimming activity were total distance travelled and the average velocity for 5 min trials. Sample sizes of 18–25 were chosen to delineate the effects of each compound from natural variability in locomotion exhibited by larvae. Touch stimuli response was conducted by gently touching the posterior portion of the tail of 20–30 larvae from each respective group and was qualitatively assessed as normal or reduced (slow response or absence of response).

### 2.6. Statistical Analysis

Statistical analysis was done using the software GraphPad Prism v.7 (San Diego, CA, USA). Swimming activity was evaluated on sample sizes ranging from 18–25 individuals, eGFP^+^ cell counts were quantified from 10–15 zebrafish, and gene expression data were collected from three pools of 10 embryos. Data are shown as the mean ± the standard error of the mean. The total distance and average velocity were analyzed using a one-way ANOVA followed by Dunnett’s multiple comparison test. vDC cell loss was analyzed using a two-way ANOVA followed by Tukey’s multiple comparison test. Gene expression data were calculated using a multiple t-test comparison with significance determined using the Holm–Sidak method. Statistical significance was determined when *p*-value < 0.05 and was indicated as * *p* < 0.05, ** *p* < 0.01, *** *p* < 0.001.

## 3. Results

Given the ambiguity in alternative neurotoxin-mediated studies, the current study aimed at providing direct comparisons on the in vivo effects of PD-inducing neurotoxins to identify the optimal candidate for DAnergic neuron ablation and motor phenotypes. All treatments were conducted starting at 3 dpf to prevent the inhibition of neurogenesis and because there is, at this time, near complete DAnergic differentiation and BBB development [12]. The varying concentrations used were to determine the optimal dose for each compound that would result in greater than 50% survival and could be used in subsequent analyses. Following the establishment of each LC_50_ concentration, DAnergic neuronal death was quantified through in vivo imaging of eGFP^+^ cells in various clusters of the vDC. These regions were chosen due to the proposed homology of this area to the nigrostriatal pathway depicted in higher vertebrate species [13,14]. In parallel, larval zebrafish exposed to each compound were examined for locomotor perturbances. Conducted under the same exposure regimen, these data will identify the most efficient of the neurotoxins to induce DAnergic ablation and motor impairment with any potential off target morphological malformations. This will serve as a platform for high-throughput pharmacological screening aimed at alleviating cellular, locomotor or morphological insults.

### 3.1. Effective LC_50_ Dose for PD Phenotypic Induction (Survival Curve/Median Lethal Concentration)

**Figure 1:**
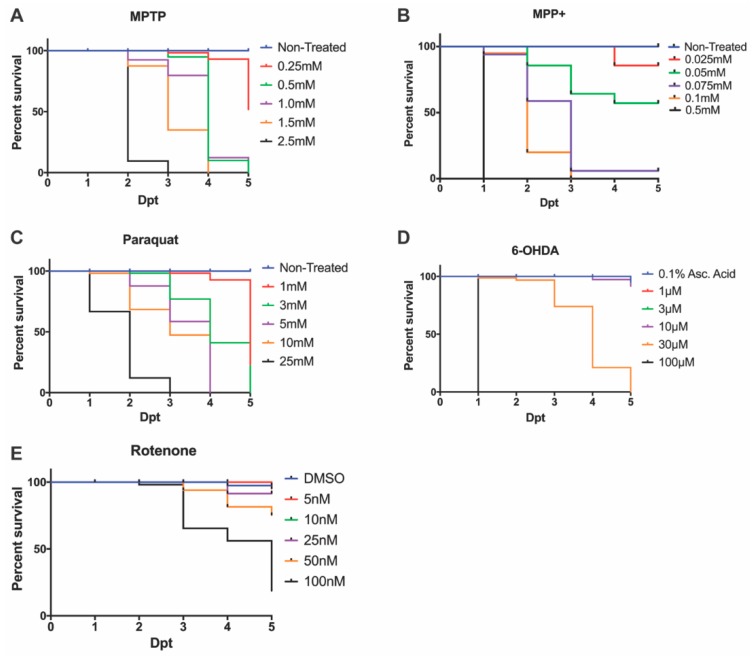
Zebrafish larvae survival following neurotoxin exposure. 3 dpf embryos were exposed to varying concentrations of (**A**) MPTP, (**B**) MPP^+^, (**C**) paraquat, (**D**) 6-OHDA, and (**E**) rotenone to determine the LC_50_ or sub-lethal dose. Lethality was quantified every 24 hpt until the zebrafish reached 7 dpf.

Following an MPTP exposure (0.25, 0.5, 1.0, 1.5 mM, and 2.5 mM), the identified LC_50_ dose to carry out further DAnergic analyses was 0.25 mM (Figure 1A). Concentrations of 2.5 mM and 1.5 mM resulted in complete mortality at 3-days post treatment (dpt) and 4 dpt respectively. In contrast, concentrations of 1.0 mM and 1.5 mM resulted in severe malformations and were shown to be completely lethal at 5 dpt. The selected concentration of 0.25 mM resulted in >50% survival, however, it is noted the mortality increased 2.5-fold from 4 dpt to 5 dpt.

**Figure 2:**
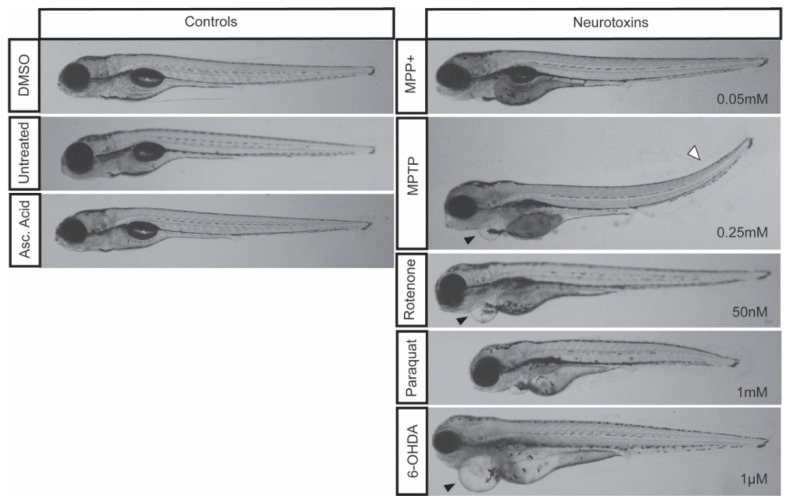
Zebrafish morphology following neurotoxic exposures. Larvae were exposed to 0.05 mM MPP^+^, 0.25 mM MPTP, 50 nM rotenone, 1 mM paraquat, and 1µM 6-OHDA starting at 3 dpf and imaged at 7 dpf. Control larvae were exposed to 0.1% DMSO, 0.1% ascorbic acid, or left untreated. Cardiac defects are depicted by a black arrowhead and a tail curvature is depicted by a white arrowhead. Refer to Table 2 for the frequency of each morphological malformation.

Upon exposure to MPP^+^, larvae treated with concentrations exceeding 0.05 mM showed 100% lethality by 7 dpf, whereas larvae treated with 0.05 mM displayed over 50% survival (Figure 1B) without severe morphological defects (Figure 2).

The herbicide paraquat was analyzed for its cytotoxic effects on DAnergic neurons due to its properties as an oxidizing agent. Exposure to 25 mM paraquat was shown to be lethal by 3 dpt, whereas, at 5 mM and 10 mM 100% lethality was observed by 4 dpt. Consequently, a concentration of 1 mM was used to examine a DAnergic loss phenotypic. This concentration contrasted with a previous report by [10], where exposure to 3 mM for two days resulted in complete mortality by 5 dpt (Figure 1C).

6-OHDA was administered at concentrations that ranged from 1 μM to 100 μM. A concentration of 100 μM resulted in 100% mortality at 1 dpt, whereas 30 μM was shown to be completely lethal by 5 dpt. Larvae exposed to concentrations of 1 μM, 3 μM, and 10 μM displayed >75% survival (Figure 1D).

Previous studies have utilized concentrations of rotenone ranging from 10 nM to 1 uM under various treatment regimens [36,38,42]. Here, we tested a range from 5 nM to 100 nM from 3 dpf to 7 dpf. The LC_50_ dose was observed to be 50 nM (Figure 1E). All further experimentation with rotenone was carried out using this dose.

### 3.2. DAnergic Neuronal Loss in the Ventral Diencephalon (vDC)

**Figure 3:**
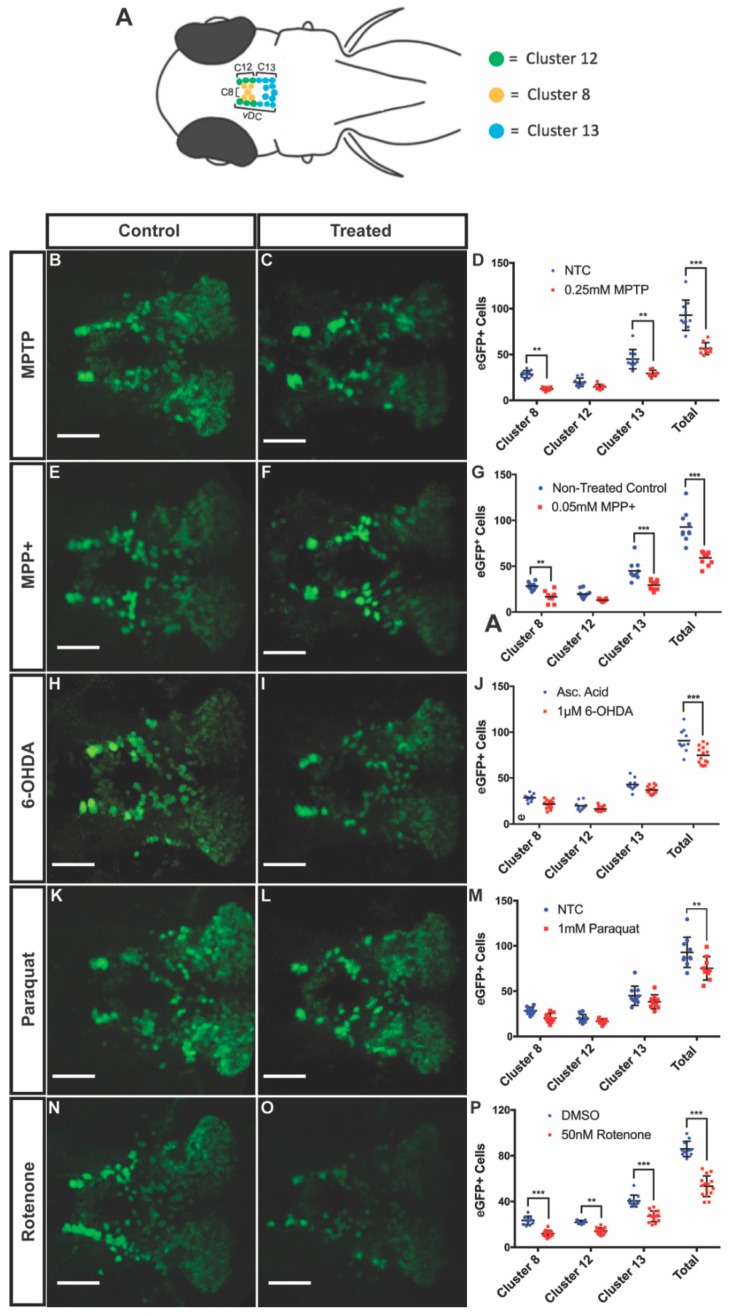
Effects of neurotoxins on ventral diencephalic (vDC) DAnergic clusters of 7 dpf Tg(*dat:eGFP*) zebrafish. (**A**) Schematic representation of eGFP neuronal clusters 8, 12, and 13 within the vDC. Cluster 8 in yellow, cluster 12 in green, and cluster 13 in blue. Dorsal view with anterior to the left. (**B**–**P**) Quantification eGFP^+^ cells within the vDC through confocal fluorescent live imaging following exposure to 0.25 mM MPTP, 0.05 mM MPP^+^, 1 uM 6-OHDA, 1 mM paraquat, and 50 nM rotenone (*n* = 9–15). All images were acquired using a maximum projection of 2µm z-stacks, scale bar = 100µm. Bars represent the Mean ± the SEM. * (*p* < 0.05), ** (*p* < 0.01), *** (*p* < 0.001).

To determine the degree of DAnergic neuronal decrease in the vDC, we treated 3 dpf larvae for four days with MPTP, MPP^+^, 6-OHDA, and paraquat at their respective LC_50_ concentrations. Following exposure to 0.25 mM MPTP, larvae displayed a 39% decrease in the total number of eGFP positive neurons in clusters 8, 12, and 13 of the vDC. We observed a 56% and 35% decrease in clusters 8 and 13, respectively, in MPTP-treated animals compared to controls, while there was no significant difference in cluster 12 (Figure 3B–D). Larvae exposed to 0.05 mM MPP^+^ exhibited similar decreases in clusters 8 and 13 with 41% and 35% reduction, respectively, while cluster 12 remained unaffected. DAnergic cells expressing eGFP were shown to decrease by 36% globally between these vDC clusters (Figure 3E–G).

In larvae treated with 1 uM 6-OHDA, there was a 19% decrease in the number of DAnergic neurons within the vDC. Interestingly, when looking at the changes within individual clusters, decreases did not reach statistical significance although there was a clear trend (Figure 3H–J).

Exposure of fish to paraquat resulted in a 18% decrease in global ventral diencephalic DAnergic neurons, however, changes in individual clusters did not reach statistical significance (Figure 3K–M).

Exposure to 50 nM rotenone was shown to significantly impact all three DAnergic clusters. Reductions of 49%, 35%, and 33% were observed following rotenone treatment in clusters 8, 12, and 13, respectively. The combined DAnergic loss cumulated to 38% within the vDC (Figure 3N–P).

### 3.3. Gene Expression

**Figure 4:**
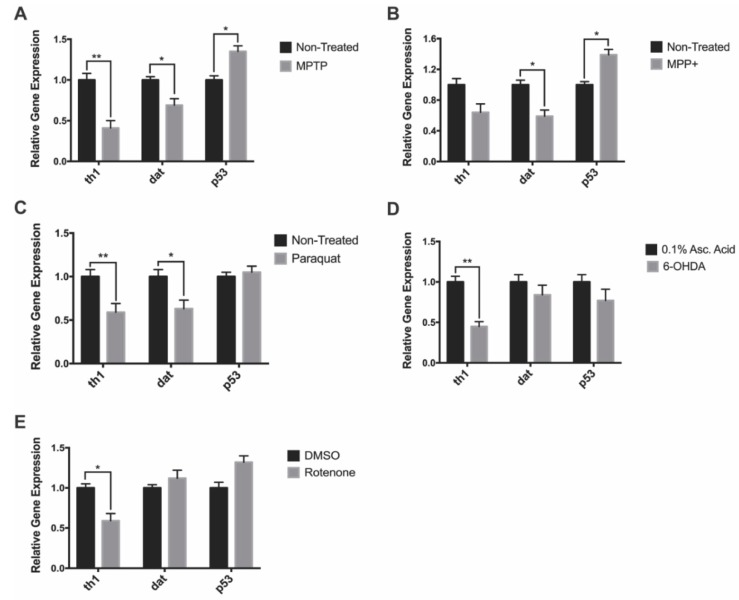
Impact of neurotoxin exposure on dopaminergic- and apoptosis-linked gene expression. (**A**–**E**) Relative expression of *dat*, *th1*, and *p53* of 7 dpf zebrafish following exposure to MPTP, MPP^+^, paraquat, 6-OHDA, and rotenone (*n* = 3 pools of 10 larvae). Bars represent the Mean ± the SEM. * (*p* < 0.05) and ** (*p* < 0.01).

We analyzed expression of genes such as *tyrosine hydroxylase 1* (gene: *th1;* protein: TH*)* and *dopamine transporter* (gene: *dat;* protein: DAT) to see if they correlated with the observed decreases in DAnergic cell numbers. In addition, we investigated whether neurotoxin exposure would trigger neuronal apoptosis through the *p53* pathway by looking at its mRNA expression. MPTP-treated larvae exhibited a 60% and 31% in *th1* and *dat* expression relative to the non-treated control, whereas, *p53* expression was shown to increase by 35% (Figure 4A). Following MPP^+^ exposure, there were 36% and 55% decreases in the expression of *th1* and *dat* respectively in treated compared to control fish. There was also a 39% increase in *p53* expression in MPP^+^ treated fish (Figure 4B). Paraquat exposure resulted in a 41% and 37% decrease in *th1* and *dat* expression respectively. However, there was no change in the expression of *p53* (Figure 4C). 6-OHDA exposure resulted in a 55% decrease only in *th1* while *dat* and *p53* expression was unaffected (Figure 4D). Finally, rotenone was shown to significantly impact *th1* expression resulting in a 41% reduction, whereas the expression of *dat* and *p53* were observed to be slightly upregulated, although changes did not reach statistical significance (Figure 4E).

### 3.4. Effects on Locomotion

**Figure 5:**
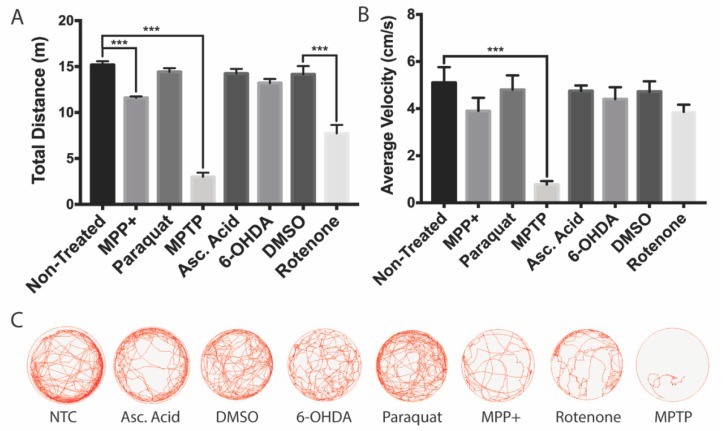
Swimming activity of zebrafish larvae following neurotoxin exposure. All zebrafish were transferred to acclimate to a 6-well plate for 15 mins prior to behavioral analyses. (**A**,**B**) Effects of MPTP, MPP^+^, 6-OHDA, paraquat, and rotenone on total swim distance and average swimming velocity (*n* = 20–25 larvae). (**C**) Overhead path images of a representative larvae for each exposure group. Bars represent the Mean ± the SEM. *** (*p* < 0.001).

To further investigate whether the decrease in DAnergic neurons within the vDC affects behavior, we analyzed the zebrafish locomotor activity. The most important reductions in both total distance and velocity were obtained for MPTP-treated larvae (80% and 85% reductions, respectively, Figure 5A,B). A milder impact on locomotion was obtained with the active metabolite MPP^+^, with only slight 24% reductions in both total distance and velocity.

Both paraquat and 6-OHDA had no significant impact on locomotion. In contrast, rotenone elicited an effect only on total distance with a 46% reduction, while the average velocity was unaffected.

## 4. Discussion

With the majority of Parkinsonian pathologies being sporadic, there lies considerable interest in the role of environmental neurotoxin exposure as a contributor of PD. While there have been studies that tested the effects of MPTP, MPP^+^ [14], 6-OHDA [43], paraquat, and rotenone [10,38] in larval zebrafish, there have been mixed and contradictory results for each drug. The majority of these studies begin treatment at 24 hpf and continue until 4 dpf or 7 dpf making it difficult to discern whether the observed reductions in DAnergic neurons result from cell death, impairment of DAnergic differentiation, or delayed neurogenesis. In this study, we conducted a direct comparative analysis between MPTP, MPP^+^, rotenone, 6-OHDA, and paraquat using the same treatment regimen to extrapolate the optimal compound that induces a decrease in DAnergic cells and a locomotor impairing phenotype. In addition, these data will emphasize the neurotoxin-mediated contribution to the onset of PD-related symptoms on zebrafish larvae.

### 4.1. MPTP and MPP^+^

The impact of MPTP on locomotion has been well characterized using in vivo models ranging from rodents [4], to amphibians [44], and medaka [45]. Similarly, in zebrafish, larval exposure to MPTP has been shown to induce DA neurodegeneration within the vDC that translates into a motor phenotype [14]. Following our treatment regimen, starting at 3 dpf to ensure a functional BBB and advanced DAnergic development, our results displayed significant locomotor dysfunction correlating with a 39% decrease in DAnergic neurons. When contrasting the effects with MPP^+^, MPP^+^ treated zebrafish showed a similar degree of DAnergic neuronal ablation, but did not display the same motor defects. MPTP requires metabolism MAO-B in the vDC to exert its neurotoxic effect, as well as the LC_50_ dose administered being 5-fold greater than MPP^+^. However, in zebrafish, the expression of MAO-B within the vDC appears to be low based on spatial distribution, thus limiting MPTP conversion to its active toxic form in this region of the brain [14]. Therefore, the perturbance in motor function shown in the current study may be a result of the constant systemic exposure to a large dose of MPTP. Furthermore, exposure to 0.25 mM of MPTP resulted in the most severe and frequent morphological defects (Figure 2).

Previous studies have shown that MPP^+^ exposure starting at 1 dpf continuing [15] for three days resulted in a decrease in DA neurons only within the diencephalon [8,14]. In addition, the decrease in DAnergic neurons was correlated with a decrease in total distance travelled [14], mean velocity and percent of time moving [46]. Our treatment regimen, starting at 3 dpf still resulted in a 36% loss in the number of DAnergic neurons. Larvae exposed to this concentration of MPP^+^ were observed to be morphologically indistinguishable from controls. Consistent with previous data, the loss of DAnergic neurons was also correlated with a decrease in total distance travelled and mean velocity. However, the decrease in distance traveled and mean velocity observed were modest compared to those obtained with MPTP. This could be due to effects of MPTP secondary metabolites, as also evidenced by the malformations observed that include the development of slight cardiac edemas and upwards tail curvatures (Figure 2).

### 4.2. Paraquat

Although paraquat is structurally similar to MPP^+^, there remains ambiguity in paraquat’s mechanism of action. It is only recently that this mechanism has been linked to genetics where one study identified three candidate genes (POR, ATP7A, and SLC45A4) whose products are required for reactive oxygen species (ROS)-mediated cell death [47]. Paraquat has been shown to cross the BBB in rats to induce dopaminergic toxicity [48], but in the current study paraquat failed to elicit the same degree of DAnergic reductions within the vDC compared to MPTP and MPP^+^. Paraquat did, however, cause a significant reduction in total eGFP expression in DAnergic cells, as well as a downregulation of *th1* and *dat* transcript levels. Moreover, paraquat had only mild effects, if any, on locomotion. Some reports document a near 3-fold difference in total distance [26], while others did not see the same deficit [10]. Contrarily to Bretaud et al. we found notable morphological defects in zebrafish treated with paraquat alone. We did not observe effects that were specific to any particular organ, but rather a reduction in the overall size of treated larvae (Figure 2).

### 4.3. 6-OHDA

Consistent with previous reports [43], zebrafish exposed to 1µM 6-OHDA showed a significant decrease of the number of DAnergic neurons in the vDC. However, this 18% reduction in DAnergic neurons did not translate into any impairment of locomotor activity. Notably, although relatively scarce, larvae exposed to 1µM were shown to develop severe cardiac malformations. These results contradict those of the study conducted by Feng et al., where they observed moderate and severe motor defects in zebrafish treated with 100 µM and 250 µM 6-OHDA respectively [43]. This effect may be a consequence of the treatment regimen adopted in that study where larvae were exposed to 6-OHDA once from 24 hpf to 5 dpf prior to the establishment of a functional DAnergic system. Furthermore, despite larval survival at higher concentrations (3 µM and 10 µM), we found that the effect of these doses on vDC DAnergic numbers to be indistinguishable from those obtained with 1µM. Nonetheless, the reduction in eGFP^+^ neurons when contrasted to MPTP, MPP^+^, and rotenone may be a consequence of the proposed neuroprotective effects of ascorbic acid [49,50].

### 4.4. Rotenone

Rotenone-induced neurotoxicity has been well documented to contribute to motor deficits in various in vivo models ranging from mice [6], rats [51], and *Drosophila* [52]. In addition, rotenone exposure has been shown to induce Lewy body inclusions and neurodegeneration in mice and rats [29,53,54]. However, rotenone exposure in zebrafish has yielded conflicting results. An initial study demonstrated that rotenone exposure of 5 µg/L and 10 µ/L starting at 24 hpf did not result in a loss of DAnergic neurons or to PD-like locomotor dysfunction [10]. In contrast, another study showed that exposure of larvae for six days starting at 24 hpf resulted in a significant decrease in the number of cells and severe mispatterning within the vDC and the olfactory bulbs [36]. Despite the decrease in DAnergic neurons, there was no major locomotor impairment. Furthermore, morphological defects associated with rotenone exposure in the study of Bretaud et al. only demonstrated a synergistic effect following co-treatment with paraquat. Here, we were able to show that exposure of larval zebrafish to 50 nM rotenone for four days starting at 3 dpf was sufficient to induce a loss of DAnergic neurons within the vDC and a mild locomotion defect. Moreover, we also observed zebrafish exposed to rotenone developing cardiac malformations (edema; Figure 2, arrowhead). Unexpectedly, we also observed a slight upregulation of *dat* following rotenone exposure. This increase may be part of a compensatory mechanism to promote the reuptake of dopamine within surviving neurons.

### 4.5. Comparison of Effectiveness between Tested Neurotoxins

Here we exposed larval zebrafish to respective neurotoxins starting at 3 dpf a time at which the DAnergic system is well established. This allowed us to test the effects of neurotoxic compounds on the survival of DAnergic neurons [55]. Using a consistent immersion treatment regiment, we were able to assess the effectiveness of neurotoxic compounds on inducing DAnergic neuronal loss and locomotor behavior. In line with previously reported studies in mammalian models [5], MPTP exposure resulted in the largest loss of DAnergic neurons. The large loss of DAnergic neurons also coincided with severe locomotion defects such as reduced total distance travelled and average velocity. Meanwhile, MPP^+^ elicited a similar degree of DAnergic ablation. However, it only perturbed motor function through total distance travel and not average velocity. Noteworthy between the two neurotoxins, fish exposed to MPTP suffered detrimental cardiac and developmental abnormalities, whereas, fish treated with MPP^+^ were indistinguishable from the non-treated controls suggesting the increased efficiency of MPP^+^ for DAnergic ablation without systemic defects in contrast to MPTP. Like MPP^+^, rotenone was shown to be effective in DAnergic ablation, while only disrupting total distance traveled, although the systemic exposure of rotenone was observed to result in apparent cardiac edemas. The effectiveness of MPTP and rotenone could be attributed to their lipophilic structure allowing for the ease of permeating the BBB [7,19,20]. This molecular nature of MPTP and rotenone is beneficial regarding our treatment regimen that uses an immersion approach. This approach relies on BBB diffusion following gill, gut, and skin absorption, and circulatory transport to the brain [15]. Moreover, 6-OHDA uptake is not specific to DAnergic neurons; the use of a norepinephrine transporter inhibitor to block noradrenergic neuron uptake could increase the specificity of neurotoxicity to DAnergic neurons in future zebrafish studies. Paraquat was the least efficient neurotoxic compound to induce DAnergic neuronal loss and locomotor defects in zebrafish. A detailed mode of action of paraquat-induced cell death remains to be defined. However, our result further supports the notion that paraquat alone may not be able to generate substantial oxidative stress to induce cell damage without increasing the exposure to a concentration that will result in significant mortality. Overall, our data suggest that under the current exposure conditions and concentrations, MPP^+^ is the most effective neurotoxin. Being the active metabolite of MPTP, MPP^+^ provides a more direct route to induce substantial DAnergic neuronal loss that translates to a locomotor phenotype without the systemic toxicity observed from MPTP exposure.

## 5. Conclusions

Despite MPTP and rotenone’s efficiency in DAnergic ablation and motor perturbation, MPP^+^ remains as the optimal compound for PD-related recapitulation. Given the ongoing issue of experimental reproducibility, the results of this comprehensive comparison will contribute to alleviate this problem and to shed light onto the extensive neurotoxin contribution to the pathology of PD. Following this exposure regimen, using MPP^+^ will provide a most effective and consistent phenotype for Parkinsonian insult that will contribute to the facilitation of therapeutic screening for cellular, motor, and morphological symptoms.

## Figures and Tables

**Table 1:** List of primers designed for qRT-PCR.

Primer	Forward Sequence (5′–3′)	Reverse Sequence (5′–3′)	Reference
*dat*	AGACATCTGGGAAGGTGGTG	ACCTGAGCATCATACAGGCG	[39]
*th1*	GACGGAAGATGATCGGAGACA	CCGCCATGTTCCGATTTCT	[40]
*p53*	ATATCCTGGCGAACATTTGG	ACGTCCACCACCACCATTTGAAC	
*ywhaz*	TCTGCAATGATGTGTTGGAGC	TCAATGGTTGCTTTCTTGTCGTC	[41]
*rpl13a*	TCTGGAGGACTGTAAGAGGTATGC	AGACGCACAATCTTGAGAGCAG	
*ef1a*	CTGGAGGCCAGCTCAAACAT	ATCAAGAAGAGTAGTACCGCTAGCATTAC	

**Table 2:** Summary of LC_50_ neurotoxic effects of MPTP, MPP^+^, paraquat, 6-OHDA, and rotenone.

Drug	LC_50_ Concentration	Locomotion	Global vDC DAnergic Loss (%)	Morphological Defects	Other Effects
MPTP	0.25 mM	Severe	39%	Cardiac and tail curvature (9/20)	Unresponsive to touch
MPP^+^	0.05 mM	Moderate	35%	None of significance	Unresponsive to touch
Paraquat	1 mM	None	16%	Stunted development (3/20)	None of significance
6-OHDA	1 µM	None	18%	Cardiac defect (2/20)	None of significance
Rotenone	50 nM	Moderate	36%	Cardiac defect (4/20)	None of significance

## Abbreviations

PD	Parkinson’s disease
hpf	Hour(s) post fertilization
dpf	Day(s) post fertilization
dpt	Day(s) post treatment
MPTP	1-methyl-4-phenyl-1,2,3,6-tetrahydropyridine
MPP^+^	1-methyl-4-phenyl-pyridinium
Paraquat	Methyl viologen dichloride hydrate
6-OHDA	6-hydroxydopamine
DMSO	Dimethyl sulfoxide
LC_50_	Median lethal dose
DAnergic	Dopaminergic
vDC	Ventral diencephalon
BBB	Blood-brain barrier
th	Tyrosine hydroxylase
dat	Dopamine transporter
eGFP	Enhanced green fluorescent protein
SEM	Standard error of the mean
MAO-B	Monoamine oxidase B
ETC	Electron transport chain
mRNA	Messenger ribonucleic acid
ROS	Reactive oxygen species
